# Dr. Lewis H. Hazeltine

**Published:** 1913-05

**Authors:** 


Dr. Lewis H. Hazeltine of Clarksville, Mich., (not listed in Polk) died in April. He was born and reared in Jamestown, N.Y.

